# Preparing for the Next Pandemic: A Cross-Sectional Study to Assess the Perspectives of Healthcare Staff and Patients on Mandatory Vaccination for Healthcare Workers in a National Health Service (NHS) Hospital Trust

**DOI:** 10.7759/cureus.91816

**Published:** 2025-09-08

**Authors:** Muhammad Shariq Rahemtoola, Anand Pillai

**Affiliations:** 1 Urology, North Manchester General Hospital, Manchester University National Health Service (NHS) Foundation Trust, Manchester, GBR; 2 Orthopaedics and Traumatology, Wythenshawe Hospital, Manchester, GBR

**Keywords:** coronavirus covid-19, guidance for next pandemic, lessons learnt, vaccination mandate, vaccine choice

## Abstract

Introduction

The United Kingdom (UK) government proposed mandatory coronavirus disease 2019 (COVID-19) vaccination for healthcare workers (HCWs) in November 2021, with a deadline to vaccinate by April 2022, after which those unvaccinated would be redeployed from patient-facing roles or dismissed from their jobs. This proposal was then revoked in March 2022, but in a future pandemic or epidemic, this may be re-implemented, and there is a lack of meaningful lessons learned in the process, as well as a lack of guidance in place for future epidemics. The perspective of the patients attending hospitals and healthcare staff (HCS) on this is unknown. We aim to gather their perspective through a questionnaire just after the 3rd wave of the COVID-19 pandemic in England.

Methods

A cross-sectional study was carried out from 4th April to 10th April 2022 in Wythenshawe Hospital in England. A questionnaire was given to HCS and patients to gather their perspective on the mandate, and the data was then analysed.

Findings

Out of 323 participants who took part in the study, 321 met the inclusion criteria and included 159 patients and 162 HCS. A total of 85 (52.5%) HCS and 70 (44.0%) patients agreed to vaccine mandates. Hundred (61.7%) HCS and 116 (73.0%) patients were of the opinion that HCS who have antibodies against the virus should have a choice. Sixty-two (38.3%) HCS and 62 (39.0%) patients agreed to a vaccine mandate for patients before admission for non-urgent care. 100 (61.7%) HCS and 83 (52.2%) patients disagreed with the redeployment of unvaccinated HCS. One hundred and five (64.8%) HCS and 74 (46.5%) patients agreed with the decision to set aside the mandate, and 99 (61.1%) HCS and 103 (64.8%) patients thought that people should continue to wear masks and maintain social distancing.

Interpretation

Almost one in two HCS and patients favour vaccine mandates for HCS. However, only one in three patients and HCS favour a mandate for patients. A large majority of participants are in favor of the choice if HCS has antibodies. More than one in two participants in both categories disagree with redeploying/dismissing unvaccinated HCS and feel that masks and social distancing should be continued. More HCS agreed to the decision to set aside the mandate than patients.

## Introduction

Coronavirus disease 2019 (COVID-19) is a highly transmissible infection caused by the severe acute respiratory syndrome coronavirus 2 (SARS-CoV-2) virus [1]. In December 2019, a cluster of viral pneumonia of unknown origin was identified in Wuhan, China [2]. This led to the first confirmed case of COVID-19 infection. The first confirmed cases in the UK were reported on 29 January 2020 [3]. The coronavirus disease was declared a Public Health Emergency of International Concern on 30 January 2020 and a pandemic on 11 March 2020 by the World Health Organization [4]. As of 1 April 2022, the UK had experienced three waves of COVID-19, with a total of 21,641,004 confirmed positive cases [5,6]. The official global death toll exceeded six million as of 6th March 2022 [3].

In the week ending 2nd April 2022, 7.60% of the population in England tested positive for COVID-19, the highest positivity rate recorded since the Office for National Statistics (ONS) study began. The hospital admission rate during that week was 20.46 per 100,000 people. In comparison, during the second wave, the peak positivity rate was 2.08%, while hospital admissions reached 36.68 per 100,000 people. Similarly, death rates during the third wave were eight times lower than those in the second wave [7]. These figures indicate that during the third wave, there was a high prevalence of COVID-19 infections in England. The dominant variant during this wave was Omicron, which has been shown to less frequently involve the lower respiratory tract and was thus less likely to cause severe disease or require hospital admission [8,9].

Another contributing factor to the reduced hospital admission and death rates during the third wave was the high vaccination rate in England [10]. Booster doses, particularly those using mRNA vaccines, were shown to reduce hospitalisation and COVID-related deaths by approximately 70% in patients infected with the Omicron variant [10,11]. The booster vaccine has been crucial in reducing disease severity and hospital admission rates associated with the Omicron variant [10,12].

On 15th March 2022, the UK government revoked the proposed requirement for mandatory vaccination among health and social care staff (HCS) [13]. This decision was made following a review of risks and benefits, including concerns regarding potential workforce shortages if the mandate were enforced [14]. This raises the question of what impact another pandemic or epidemic or a new dominant variant could have on an already weakened healthcare system in the UK, and whether COVID-19 or any other vaccination or booster doses against other infectious diseases should be made mandatory, especially for healthcare workers (HCWs) who care for vulnerable and high-risk patients on a daily basis. Another question is what meaningful lessons were learnt in the process of the mandate and what guidance can be put in place for future epidemics or pandemics from the lessons learnt and perspectives of introducing a vaccine mandate.

In view of the above, and given the possibility that the requirement could be reintroduced in the event of another epidemic or global pandemic, we aimed to assess the perspectives of patients and healthcare staff on mandatory COVID-19 vaccination for HCWs. We conducted a cross-sectional study in April 2022, shortly after the third wave of COVID-19 in England. The findings may help inform future healthcare policy decisions during public health emergencies.

A cross-sectional study was carried out in a tertiary hospital. Patients attending the Accident & Emergency (A&E) and Fracture Clinic were asked to fill in a questionnaire to gather their perspective on COVID-19, vaccination, and the vaccine mandates for HCW. The same questionnaire was used to gather the hospital staff's perspective. The responses to the questionnaire were then analysed to determine the attitude of the patients and staff towards mandatory vaccination in that given population. The study included both HCWs and patients, as HCWs are the population on which the mandate was implemented, and patients are the population most at risk and affected by the decision of having vaccinated or unvaccinated HCWs, especially through a mandate.

## Materials and methods

Study design

This cross-sectional study was carried out to determine the attitudes of HCS towards vaccine mandates for HCWs. A questionnaire consisting of 19 questions was developed for this study. Of these, 14 were binary questions with “Yes” or “No” as the response options. The remaining five questions were open-ended and aimed at exploring participants' thoughts and perspectives. The questionnaire used is included in Appendix 1. The same questionnaire was used for both patients and HCS. Data collection was conducted from 4th April 2022 to 10th April 2022 at a tertiary Hospital in Manchester.

Literature review

For the literature review, PubMed was used as the primary search engine to access the MEDLINE database. The key search terms used to identify suitable literature on the topic included, but were not limited to: COVID-19, vaccination, mandates, hospitalisation, death rate, SARS-CoV-2, transmission. The main journals used for reference were The Lancet, The BMJ, and The New England Journal of Medicine (NEJM).

Sample size

A total of 323 participants took part in the study. Of these, 321 met the inclusion criteria. Among the eligible participants, 159 were patients attending either the Fracture Clinic or the Accident and Emergency (A&E) waiting areas. The remaining 162 were HCS.

Inclusion criteria

For this study, participants were divided into two categories, namely HCS and patients. The inclusion criteria for HCS consisted of an age of 18 years or above and HCS working in any department within Wythenshawe Hospital. HCS was defined as anyone who selected the option of an HCS on the questionnaire, and this included doctors, nurses, healthcare assistants, patient-facing staff, including, allied workers, receptionists, and medical students. The inclusion criteria for patients consisted of an age of 18 years or above, and participants present in the above-mentioned hospital outpatient departments as a patient or accompanying a patient to the hospital during the study period.

Data analysis 

Data were analysed using a descriptive analysis. Each questionnaire item was analysed separately for healthcare workers and patients. Responses were expressed as frequencies and percentages, and the results were described narratively. No inferential statistical tests were applied, as the aim of the study was exploratory and focused on identifying the distribution of views across the two groups on the perspectives of a vaccine mandate on HCWs.

## Results

Results

The data collected from the questionnaire were recorded in the form of Table 1 below. A total of 159 patients (female: 59.1%; male: 40.9%) and a total of 162 HCS (female: 61.1%; male: 38.9%) participated and met the inclusion criteria.

**Table 1:** Summary of the data collected from the questionnaires HCW: Health care worker

Category	Patients	Healthcare Staff (HCS)
Tested Positive for COVID-19	49.7% (n=79)	67.3% (n=109)
Family/Close Circle Tested Positive	83% (n=132)	87.7% (n=142)
Hospitalisation (Participant)	2.7% (n=4)	3.1% (n=5)
Hospitalisation (Close Contact)	19.5% (n=31)	9.9% (n=16)
COVID-19 Related Death in Family/Circle	16.4% (n=26)	14.2% (n=23)
Hospital-Acquired COVID-19	12.6% (n=20)	12.3% (n=20)
Believe Vaccine is Safe	79.9% (n=127)	93.8% (n=152)
Believe Vaccine is Unsafe	9.4% (n=15)	1.9% (n=3)
Unsure About Vaccine Safety	10.7% (n=17)	4.3% (n=7)
Fully Vaccinated (Double Dose)	91.2% (n=145)	98.8% (n=159)
Received or Planning Booster Dose	78% (n=124)	92.6% (n=150)
Preferred Pfizer/BioNTech Vaccine	34.6% (n=55)	60.5% (n=96)
No Vaccine Preference	54.1% (n=86)	33.3% (n=54)
Preferred AstraZeneca Vaccine	7.5% (n=12)	5.6% (n=9)
Preferred Moderna Vaccine	3.8% (n=6)	0.6% (n=1)
Support Government Vaccine Mandate for HCWs	44% (n=70)	52.5% (n=85)
Oppose Government Vaccine Mandate for HCWs	36.5% (n=58)	32.1% (n=52)
No Opinion on Government Vaccine Mandate	19.5% (n=31)	15.4% (n=25)
Support Exemption for Naturally Immune HCWs	73% (n=116)	61.7% (n=100)
Oppose Exemption for Naturally Immune HCWs	22.6% (n=36)	36.4% (n=59)
Support Mandate for Patient Vaccination Before Non-Urgent Admission	39% (n=62)	38.3% (n=62)
Oppose Mandate for Patient Vaccination	55.3% (n=88)	59.3% (n=96)
Support Redeployment of Unvaccinated HCWs	41.4% (n=66)	35.8% (n=58)
Oppose Redeployment of Unvaccinated HCWs	52.2% (n=83)	61.7% (n=100)
Support Revoking Vaccine Mandate	46.5% (n=74)	64.8% (n=105)
Oppose Revoking Vaccine Mandate	44.7% (n=71)	32.7% (n=53)
Support Continued Masking and Social Distancing	64.8% (n=103)	61.1% (n=99)
Gender (Female)	59.1% (n=94)	61.1% (n=99)
Gender (Male)	40.9% (n=65)	38.9% (n=63)

COVID-19 exposure and impact

Seventy-nine (49.7%) patients and 109 (67.3%) HCS reported having previously tested positive for COVID-19. One hundred and thirty-two (83.0%) patients and 142 (87.7%) HCS stated that a family member or someone in their close circle had tested positive. In terms of hospitalisation, four (2.7%) patients and 31 (19.9%) of their close contacts had been hospitalised. With regard to HCS, five (3.1%) and 16 (9.9%) of their close contacts were hospitalised. Twenty-six (16.4%) patients and 23 (14.2%) HCS reported a death in the family or close circle due to COVID-19. With regard to hospital-acquired COVID-19 infection, 20 (12.6%) patients and 20 (12.3%) HCS responded that either they or someone in their close circle contracted COVID-19 while in hospital for other treatments or work.

Perceptions of vaccine safety

It was noted that 152 (93.8%) HCS and 127 (79.9%) patients believed that COVID-19 vaccines are safe. Three (1.9%) HCS and 15 (9.4%) patients considered the vaccines unsafe. The remaining seven (4.3%) HCS and 17 (10.7%) patients were unsure about the safety of the vaccine. The most common comment on the safety of the vaccine was ‘UNSURE’.

Vaccine uptake and booster status

With regard to vaccination, 159 (98.8%) HCS were fully (double) vaccinated in comparison to 145 (91.2%) patients. Booster uptake was lower, with 150 (92.6%) HCS and 124 (78.0%) patients having received or planning to receive a booster dose.

Vaccine preference

In terms of preference for a vaccine, Pfizer/BioNTech was the most preferred in HCS (n=96, 60.5%), while 54 (33.3%) HCS did not have a preference. However, in the patient cohort, more than half of the cohort (n=86, 54.1%) had no preference, while only 55 patients (34.6%) preferred Pfizer/BioNTech. AstraZeneca was preferred by 12 (7.5%) patients and nine (5.6%) HCS, while Moderna was least preferred in both groups, with six (3.8%) patients and one (0.6%) HCS choosing it as preferred vaccine.

Vaccine hesitancy

As to the reasons for not taking the vaccine, participants from both groups raised concerns regarding long- and short-term side effects. Also, a low perceived risk of infection was noted in both groups who declined the vaccine. There were also comments on natural immunity from infection; vaccines' inability to prevent infection, vaccines having been ‘rushed’, and concerns on their safety as well as impact on fertility. Other concerns from patients with regard to vaccines were mainly regarding the safety for children; ‘vaccines are in trial or experimental phase’ and ‘long-term effects’. In addition to the concerns mentioned above, three HCS were concerned about the impact on the menstrual cycle, and one HCS believed that the vaccines caused miscarriages. There were also concerns in HCS about the need for regular booster doses. 

Reported side effects 

The most common reported side effects in both participant groups were ‘flu-like symptoms, head and body aches, sore arm, and fatigue. Two HCS reported irregularities in their menstrual cycle; one HCS reported ‘frozen shoulder’, and 1 HCS reported that the vaccine ‘has weakened their immune system’. One patient reported ‘stroke-like symptoms’ after the vaccine, and another patient reported a ‘heart-related condition’.

Perspective on the government vaccine mandate policy for HCWs

With regard to the government policy for vaccine mandates, 85 (52.5%) HCS supported the mandate and 52 (32.1%) opposed it. The remaining 25 (15.4%) did not express an opinion on this question. Among patients, 70 (44%) agreed to the mandate while 58 (36.5%) disagreed. Thirty patients (19.5%) did not express an opinion on this question. 

Perspective on exemption for naturally immune HCWs

Hundred (61.7%) HCS and 116 (73%) patients are of the opinion that HCS who have antibodies against COVID-19 should have the choice to receive or decline the vaccine (opt out). Fifty-nine (36.4%) HCS and 36 (22.6%) patients were against this exemption. The remaining participants gave no response to this question.

Vaccine mandate for patients for non-urgent hospital admissions

Sixty-two (38.3%) HCS and 62 (39%) patients supported mandatory vaccination for patients prior to non-urgent hospital admission, unless contraindicated. Ninety-six (59.3%) HCS and 88 (55.3%) patients opposed this.

Redeployment of unvaccinated HCWs

In terms of redeployment of unvaccinated HCWs from patient-facing roles, 58 (35.8%) HCS and 66 (41.4%) patients supported redeployment from patient-facing roles. 100 (61.7%) HCS and 83 (52.2%) patients opposed the redeployment of HCWs.

Perspective on revoking the mandate

It was also observed that 105 (64.8%) HCS and 74 (46.5%) patients agreed with the decision to set aside the vaccine mandate for HCS. On the other hand, 53 (32.7%) HCS and 71 (44.7%) patients disagreed.

Continued use of masks and social distancing

Ninety-nine (61.1%) HCS and 103 (64.8%) patients supported continued mask-wearing and social distancing measures.

## Discussion

Literature review

SARS-CoV-2 Virus

Current literature suggests that the SARS-CoV-2 virus is transmitted mainly through respiratory droplets and aerosols [15]. Therefore, the use of face masks has been essential in preventing the spread of the disease, and this has been well demonstrated in multiple studies conducted throughout the pandemic [16]. At present, it is unclear whether the virus can be transmitted through other routes [17-19].

Basic Reproduction Rate (R₀)

Most studies, including the WHO estimates, have indicated that the R₀ of COVID-19 is around 2.0 to 3.0 [20,21]. This implies that the virus is highly transmissible. It is therefore very important to identify positive cases to reduce the spread, especially in healthcare settings.

Case Fatality Risk

In the second wave of the epidemic in England, the overall risk of fatality among those diagnosed with COVID-19 was 1.4% [22]. The highest case fatality risk (CFR) was seen amongst those over 80 years old (17.2%; women: 13.2%, men: 24.8%), followed by those aged 70-79 years (9.7%; women: 6.7%, men: 12.8%) [22]. Overall, in all age groups, men had higher death rates than women [22]. Also, studies have shown that obesity, diabetes, and chronic kidney disease are important risk factors for a poor outcome [23,24].

COVID-19 Vaccine Uptake

Several vaccines have been developed to protect against severe COVID-19 disease. Currently, three vaccines are being used in the UK - Moderna, Oxford/AstraZeneca, and Pfizer/BioNTech [25]. Around 49.6 million people have been double vaccinated, representing 73.7% of the UK population, and 39 million people have received a booster dose [26].

Efficacy of Vaccines

It has been observed that vaccinated individuals tend to be more likely to be infected with Omicron rather than other variants such as Delta [27]. Furthermore, studies have demonstrated that the COVID-19 vaccines approved in the EU are effective in preventing severe COVID-19 disease and reducing hospitalisation rates [27]. In fact, trials evaluating Pfizer/BioNTech demonstrated a 95% efficacy at preventing severe COVID-19 [28,29]. Moderna demonstrated an efficacy rate of 94.1% in trials, while AstraZeneca was between 91.3% and 95% in preventing severe disease [29,30].

Booster Dose

It has been observed that over time, the efficacy of vaccines reduces as the antibody level decreases [27]. The average time over which vaccine protection lasts is around six months, after which booster doses are needed to prevent a drop in immunity [27].

Vaccine Safety

The vaccines approved for use in the UK are continuously monitored for safety, and a monthly report of side effects is analysed [31,32]. The WHO and the MHRA of the UK have reviewed the side effect profiles and overall impact of all vaccines licensed in the UK and have deemed them safe for use [31,32].

Vaccine Mandates

The UK government initially required mandatory vaccination for health and social care staff by March 31, 2022, but this was revoked on March 15, 2022 [13]. However, the debate about mandatory vaccination for HCWs against COVID-19 remains relevant as the situation is continuously evolving, and another wave of the virus may bring the legislation back. In September 2021, when the consultation regarding mandatory vaccination began, 110,004 NHS staff were unvaccinated, representing 7.6% of the workforce [33,34]. As of January 16, 2022, 80,092 NHS staff (5.4%) remained unvaccinated, and the government predicted around 5% would still be unvaccinated by April 1, 2022 [33,34]. Had the regulation not been revoked, these 5% would have been redeployed or dismissed for refusing the vaccine, potentially with devastating consequences for the NHS [33,35]. The Royal Colleges of Obstetricians and Gynaecologists, General Practitioners, Midwives, and Nursing, respectively, raised concerns about the impact of vaccine mandates on services due to workforce losses in an already understaffed system and called for the rule to be deferred [36].

Discussion

We have seen that COVID-19 was found to be more prevalent among HCS and their families or close contacts than among patients (HCS: 67.3%; patients: 49.7%). This suggests that HCS are more at risk of contracting COVID-19 and thereby may be more likely to transmit the infection. Notably, the rate of hospital-acquired COVID-19 infection was similar between HCS (12.3%) and patients (12.6%). Research has shown that super-spreading events are common in healthcare settings, particularly during waves of infection [37]. In fact, during the first wave in the UK, 11.93% of hospitalised COVID-19 patients acquired the infection whilst admitted [38]. These figures are significant and are often used to advocate for a vaccine mandate for HCWs. 

In terms of the vaccine safety perception, a higher percentage of HCS believed the vaccine to be safe compared to patients (HCS: 93.8%; patients: 79.9%). Interestingly, more than 90% of participants in both groups were double vaccinated, suggesting that some individuals who had concerns about safety still chose to be vaccinated. However, hesitancy around the booster dose was more notable, especially amongst patients, where 22% of patients and 7.4% of HCS either had not received or were unwilling to receive the booster. These figures are of concern, as evidence has shown that vaccine efficacy reduces over time. People who have had the booster dose are less likely to develop severe illness or death from the infection as compared to people who were only double vaccinated.

The reasons for vaccine hesitancy identified in our study mirror those seen in national and international literature. Vaccine hesitancy has persisted throughout the pandemic among both the general population and healthcare workers [39,40]. This is particularly worrying, as hesitancy among HCS may influence public attitudes and reduce vaccination uptake further [39]. The reasons for hesitancy are complex and multifactorial, including skepticism, low perceived personal risk, fear of side effects, fertility concerns (especially among younger women), the rapid development and approval of the vaccines, delays in personal decision-making, and religious or cultural beliefs [41-47].

Concerns regarding side effects had been one of the leading reasons for hesitancy, despite the fact that major health institutions had deemed the vaccines as safe [31,32]. A study conducted in Germany in May 2021 revealed that 70% of respondents would be more likely to accept a vaccine if it had no side effects [48]. Another reason for vaccine hesitancy has been the concern that mRNA vaccine technology is new, and the long-term effects are unknown [49]. Traditionally, vaccines have relied on inactivated virus, live attenuated virus, or antigenic proteins. The mRNA vaccine has not been conventionally used because RNA is labile and is rapidly disintegrated by ribonucleases [49]. Pfizer/BioNTech and Moderna vaccines have mitigated this drawback using modified mRNA and lipid nanoparticles [49]. These compounds have already been used in FDA-approved chemotherapies, vaccines, anti-fungals and analgesics [49]. Long-term negative effects of mRNA vaccines are considered highly unlikely, given that it has a short half-life of a few days and do not integrate into host chromosomes [49,50].

With regards to concerns on fertility and miscarriage in women, current evidence does not support any link between COVID-19 vaccination and impaired fertility [51-53]. The vaccine has been clinically approved as safe and effective for most of the population [51-53]. Most side effects reported by participants in our study have aligned with the national data in the general population and are recognised and acceptable side effects of the vaccine [54]. The concerns about changes in the menstrual cycle reported in the study have also been widely reported in the UK, with more than 30,000 reports of these events. However, most of these people have reported that the change in their menstrual cycle was temporary, and there is no evidence of any permanent change to the menstrual cycle or fertility [52].

It was also noted that Pfizer/BioNTech was the most preferred vaccine amongst both HCS and patients. One of the reasons for this could be because it was one of the widely available vaccines in the UK. Also, Pfizer/BioNTech has been among the first vaccines developed against COVID-19 and has shown to have a high efficacy (95%), and this was widely mentioned in the mass media [28].

With respect to vaccine mandates, approximately one in two HCS and 44% of patients supported mandatory vaccination for HCWs. In contrast, only 38.3% HCS and 39% patients supported mandates for patients prior to hospital admission for non-urgent care. This shows that the majority of HCS and patients feel that the vaccine mandates for HCS are more essential than for patients. This could be due to the perceived responsibility of HCS in protecting vulnerable individuals. Additionally, many participants emphasized the right of patients to be admitted without discrimination and to have access to care, regardless of vaccination status.

In terms of the HCS mandates, the study shows that the views of the participants are mixed and divided, and this means that compliance with a mandate may be difficult. Studies have shown that mandatory vaccination encourages people who are hesitant to come forward and get vaccinated, and hence protects the vulnerable population. However, the context of the country involved and pre-mandate vaccination levels are essential in determining the impact of vaccine mandates [55]. In the UK, the proposed vaccine mandate for HCS increased uptake by only 2.2% [33,34].

One rationale for mandating vaccines among HCS is the professional obligation to "do no harm." Unvaccinated HCS may pose a greater risk of transmitting infection to vulnerable and high-risk patients [56]. This is one of the key reasons for justifying vaccination of HCS or showing immunity against occupational infections such as hepatitis B, measles, and rubella [56]. However, it is important to note that vaccinated people can still contract and transmit COVID-19. The basic reproduction rate for vaccinated people in comparison to unvaccinated is still unclear, and early studies have shown an almost equal rate in both categories [27,57]. This raises questions about the effectiveness of mandates in reducing transmission.

A majority of participants in the study (61.7% HCS; 73% patients) believed that HCS who have antibodies against COVID-19 should have the choice to receive or decline the vaccine. This shows that the participants feel that the choice of HCS is essential, especially if they already have some form of natural immunity or protection. However, the duration and extent of protection conferred by antibodies remain uncertain. Before the mandate was revoked, the UK government's policy included redeploying or dismissing unvaccinated HCS. We noted that only a minority of participants in our study (35.8% HCS and 41.4% patients) supported this measure. However, more patients than HCS agreed with redeployment or dismissal, and this may be because more patients believe that unvaccinated HCS carry more risk of transmitting the virus and thereby pose a risk to patient safety.

More participants in both groups supported the revocation of the mandate than supported the mandate itself. This suggests a general preference for choice rather than enforcement in both participant groups. This could also be because participants did not approve of the dismissal of staff in an already constrained healthcare setting and preferred for the policy to be revoked. It is important to note that several participants who agreed to the vaccine mandate also agreed to the decision to revoke the policy. When interviewed, some participants mentioned that the policy was not well planned. Others mentioned that although they were happy to comply with the mandate, they would not want other HCS to lose their jobs because of the mandate. These attitudes reflect the broader debate between public health obligations and individual rights. The argument made against mandatory vaccines by participants is also about the human rights of HCS and the freedom of choice.

It was also seen that around 60% of HCS and patients were of the opinion that masks and social distancing should be continued. This shows that most participants understand the importance of masks in preventing the spread of the infection and believe it to be essential during an epidemic or pandemic. Some participants also mentioned that they should be continued only in healthcare settings and when dealing with high-risk individuals. This suggests a high level of awareness about non-pharmaceutical interventions and their role in preventing the transmission of COVID-19. 

The view of HCS and patients on a vaccine mandate mentioned above is important as this will help to guide further decisions around a mandate, especially in crisis and emergency situations. However, it is important to note that the proposed vaccine mandate for HCS increased uptake of the COVID-19 vaccine by only 2.2% in this study. This is of significance as it shows that the compulsory implementation of a mandate with a lack of consultations with stakeholders is bound to meet with resistance and is unlikely to give significant benefit to the mandate. It is important to mention that the government did revoke the proposed mandate which could be explained from the low intake from the mandate and increased herd immunity due to already high vaccine uptake in England.

Unfortunately there is a lack of meaningful lessons learned in the process given the rapid implementation, lack of consultations and followed by revoking the mandate. There is also a lack of guidance in place for future epidemics or pandemics for governments and institutions to act on, and it is still uncertain how the general population, including HCWs and patients, will respond to a mandate in the future. 

Limitations

The study was limited to a single NHS Hospital Trust, which limits its generalizability. Participation was voluntary, so people with stronger views on the topic may have been more likely to take part and thereby be over-represented. Another limitation of this study affecting its generalisability is that only a descriptive analysis was performed; no inferential statistical tests were applied. As such, while the results highlight trends and differences in perspectives between groups, they cannot establish whether these differences are statistically significant or generalizable. Furthermore, there is potential for selection bias, as the study population was drawn from a single NHS Trust, which may not be representative of all healthcare workers and patients in other settings. We therefore recommend further cross-sectional studies across multiple Trusts, including primary, secondary, and tertiary care centres, to capture the perspectives of a broader and more representative study population. However, this may be challenging to achieve given that the acute phase of the pandemic has passed.

## Conclusions

Although vaccination is essential and has been shown to save lives, this study highlights that the concept of a vaccine mandate remains highly debated among both patients and healthcare staff (HCS). In these circumstances, and with a lack of clear evidence on whether vaccines prevent or reduce the transmission of COVID-19, it seems difficult to impose a mandate, and any such policy is bound to meet with resistance. A feeling of infringement on fundamental human rights could even further reduce HCS and public trust in governments and affect the support for vaccination and other public health campaigns. We believe that broader consultations are essential with stakeholders before such policies are brought forward. The potential dismissal or redeployment of unvaccinated staff could be especially harmful in an already workforce-constrained healthcare system, with possible consequences for patient safety and service delivery. It is also noteworthy that vaccine uptake has been high in the UK, and at the time of this study, more than 90% of participants were double vaccinated. Education on the importance of vaccination, especially the booster dose, is essential and may be more productive in reducing vaccine hesitancy and dismissing false news on vaccines and their side effects than a mandate.

## Figures and Tables

**Figure 1:**
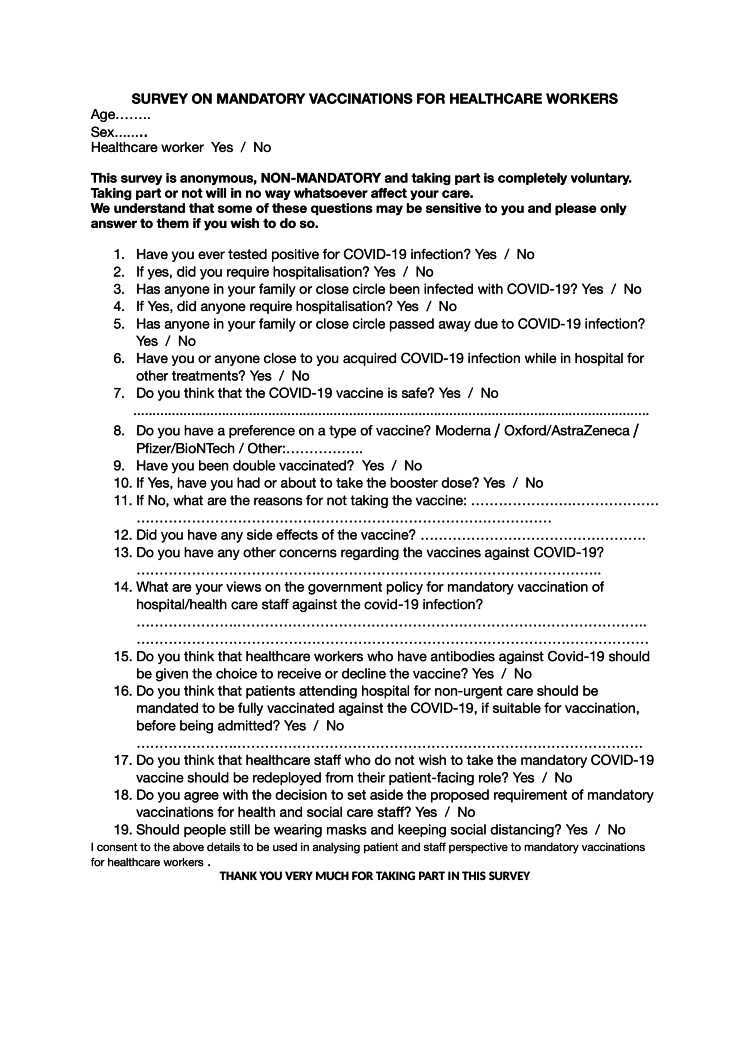
Survey questionnaire used Appendix 1 The same questionnaire was used both for patient and HCS.

## Appendices

The questionnaire used is included in Appendix 1 below.
